# Long-term effects of a ten-year osteoporosis intervention program in a Swedish population—A cross-sectional study

**DOI:** 10.1016/j.pmedr.2016.12.024

**Published:** 2017-01-02

**Authors:** Ann-Charlotte Grahn Kronhed, Helena Salminen

**Affiliations:** a Rehab Väst, Vadstena, Primary Health Care Centre, Local Health Care Services in the West of Östergötland, Sweden; b Social Medicine and Public Health Science, Division of Community Medicine, Department of Medical and Health Sciences, Linköping University; c Division of Family Medicine, Department of Neurobiology, Care Sciences and Society, Karolinska Institutet, Sweden

**Keywords:** Bone mineral density, Community-based, Fall, Fracture, Osteoporosis, Prevention

## Abstract

The aim of the study was to explore long-term effects seven years after the completion of a ten-year community-based osteoporosis intervention program in Vadstena, Sweden. The association between calcaneal bone mineral density and several life style factors, and the impact of risk factors for sustaining a fracture after the age of 50 were also studied. Previous participants in the intervention group, and matched subjects were invited to calcaneal bone mass measurement by a portable device including the dual X-ray and laser (DXL) technology by Calscan, and to complete a questionnaire in 2006. A total of 417 persons (63% of those invited) in the intervention (I) group, and 120 persons (47% of those invited) in the control (C) group participated. Mean age was 63 years (37–94 years). There was somewhat more knowledge of osteoporosis in the I-group (M = 18) than in the C-group (M = 17) (*p* < 0.05), and more use of shoe/cane spikes in elderly women in the I-group (67%) than in the C-group (40.5%). The fully adjusted model of logistic regression showed that participants with an osteoporotic DXL T-score (≤− 2.5) had a 3-fold increased risk (95%CI 1.48–6.89) of having a history of a self-reported fracture after the age of fifty compared to women with a calcaneal T-score >− 2.5. The long-term effects of a ten-year, community-based, osteoporosis intervention program on knowledge and behavior were modest seven years after its completion.

## Highlights

• The effect of community-based osteoporosis intervention seems modest in the long run.
• Elderly women were compliant with fall prevention advice using spikes in winter.
• An osteoporotic calcaneal T-score may imply increased risk for fracture.
• Moderate/high physical activity was positively associated with calcaneal BMD in men.
• Brisk walks in the summer were positively associated with calcaneal BMD in women.

## 1 Introduction

Osteoporosis and fragility fractures are major, worldwide health problems. Sweden has one of the highest incidences of osteoporotic fractures in the world (Kanis et al., 2002). Primary healthcare has the main responsibility for the prevention of osteoporosis and fragility fractures, as stated by WHO (Kanis and on behalf of the World Health Organization Scientific Group, 2007), and community-based intervention strategies are needed to address the problem. The lifetime risk for a hip, spine or forearm fracture at the age of 50 is estimated at 46% for women, and 22% for men, in Sweden (Kanis et al., 2001). The mean age of hip fracture is 80 years for women, and 76 years for men, in Sweden (Johnell and Kanis, 2005). The costs of hip fractures are high for the society and has been estimated at approximately US $21,000 for the first year (Hernlund et al., 2013). High morbidity and increased mortality after hip fracture have been seen in many studies. About 20–30% of hip fracture patients die during the first year after hip fracture (Kanis et al., 2003).

Low bone mineral density (BMD) is a strong risk factor for fractures. The T-score is defined by WHO as a BMD value, minus the mean value for a reference group of “young adult women”, divided by the standard deviation (SD) for this reference group. A T-score measured by dual energy X-ray absorptiometry (DXA) equipment at the spine, hip or midradius sites, of not more than 1 SD below the mean value of peak bone mass in young adult women, is defined as normal. A T-score value greater than 1 SD below the young adult mean, but less than 2.5 SDs below this value, is defined as osteopenia, and a T-score of 2.5 SDs or more below the mean is defined as osteoporosis (Anon, 1994). Peripheral measurements of BMD are not recommended for diagnostic purposes (http://www.iscd.org/), but all measurements sites can be predictive of fractures, and may be used for risk estimation (Cummings et al., 1993, Brismar et al., 2010). Stationary DXA equipment, as well as the portable dual X-ray and laser (DXL) equipment, are used as tools for fracture risk estimation. BMD of the calcaneus, as measured by the DXL, has shown good predictive ability for hip fractures in a prospective study of Swedish women (Brismar et al., 2010). A recently published prospective study of hip fractures in elderly women that compared DXL scans of the calcaneus with DXA scans of the hip, including the FRAX tool *(*www.shef.ac.uk/FRAX*)*, showed that BMD of the calcaneus had a fracture-predictive ability similar to BMD of the femoral neck, measured by DXA and FRAX (Lundin et al., 2015).

A ten-year community-based intervention program “the Vadstena Osteoporosis Prevention Project (VOPP)”, was carried out in Vadstena community, Sweden 1989–1999. The health-education program addressed personnel working at schools, kindergartens, social welfare offices, nursing homes and municipal home-help service units, retired people's associations, study circles, sports clubs, grocery stores, larger companies and catering services. Collaboration was established with shops to encourage the use of good lighting, sturdy shoes, and spikes. Walking, weight-bearing and balance training groups were initiated. A gymnasium with sequence training equipment was expanded at the primary health care centre (PHCC). Checklists of environmental hazards for osteoporosis and falls were distributed via the pharmacy and the PHCC. The individual intervention comprised a personal letter to the participants in the intervention group and included a health profile and a note with specific advice about how to decrease possible risk behavior for osteoporosis. Individuals aged 65 years and over also received written advice concerning how to make their living environment safer (Waller et al., 1997, Waller et al., 2002, Grahn Kronhed et al., 2006, Grahn Kronhed et al., 2005). The ten-year intervention program showed no effect between the intervention and the control groups concerning knowledge of osteoporosis (27 questions) (Waller et al., 2002). Elderly participating at follow-ups in the intervention group reported more use of shoe/cane spikes and more moderate level of physical activity than the control group (Grahn Kronhed et al., 2006). Forearm fracture incidence decreased in women in the intervention area, but the decrease was not found in the control area (Grahn Kronhed et al., 2005).

The main aim of the present cross-sectional study was to explore effects of a ten-year, community-based, osteoporosis prevention program seven years after the completion on the following parameters; calcaneal BMD, life style factors, and the impact of risk factors for sustaining a fracture after the age of 50.

## 2 Methods

### 2.1 Context

The core of the ten-year VOPP intervention program was health education to residents of all ages in order to increase awareness of risk factors for osteoporosis, falls and fractures using community-based and individual intervention strategies (Waller et al., 1997, Waller et al., 2002, Grahn Kronhed et al., 2006, Grahn Kronhed et al., 2005). An extension of the original intervention program was initiated in the 2000s and carried on until 2006. This phase implied a more high-risk strategy for those with presumed osteoporosis. A nurse at the PHCC carried out medical check-ups by DXL calcaneal measurements and a questionnaire. Those with low BMD-values and risk factors for falls and fractures were referred to a physician and/or a physiotherapist.

#### 2.1.1 Subjects

**Fig. 1:**
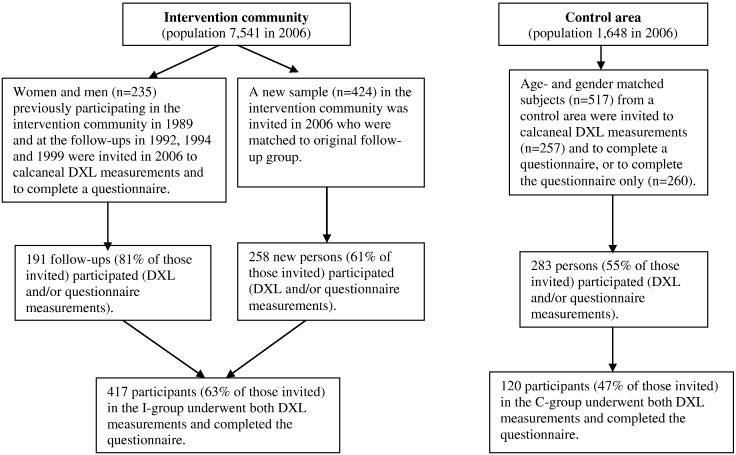
Flowchart of participants in the intervention (I) and the control (C) groups (Vadstena; Kvarnholmen/Kalmar city centre, Sweden in 2006).

A quasi-experimental design was used for the original study. Vadstena the intervention community, and the new matched control area of Kvarnholmen in the Kalmar city centre, are both located in southern Sweden. The control area was selected for its similarity to the study population's age structure, with many elderly people in both areas. Participants were selected from population registries and randomised in 2006. Three groups were invited for measurements in 2006:

1) Follow-up participants from random samples participating in the VOPP 1989, 1992, 1994, and 1999 (*n* = 235) were invited to calcaneal DXL measurements, and to complete a questionnaire.
2) Persons matched to the follow-up group were invited in Vadstena (*n* = 424) for calcaneal measurements and to complete a questionnaire.
3) Age and gender matched persons from the control area (*n* = 517) were invited, where one subgroup (*n* = 257) was offered calcaneal measurements and requested to complete a questionnaire, while another subgroup (*n* = 260) was requested to complete the questionnaire only (Fig. 1). In the present study, those participants who underwent both calcaneal measurements and completed the questionnaire were included.

#### 2.1.2 Test procedure

A questionnaire was mailed to the participants along with a written invitation to bone mass measurements. The questionnaire comprised 74 general questions concerning lifestyle (physical activity levels, smoking and drinking habits, and nutrition), diseases, medication, prior fractures (in adulthood), parents' fractures, and height as young adults. Three questions dealt with the respondent's current level of physical activity. One question concerning physical activity levels at work was rated by four activity grades with respect to skeletal loading (Saltin and Grimby, 1968). The lowest grades 1–2, were called the “light level”, and the highest grades 3–4 the “heavy level”. Levels of leisure time physical activity were classified by a six-grade scale. Previous evaluation suggests that this scale has acceptable validity in specific for older adults. Grades 1–2 designated a “low level”, grades 3–4 designated a “moderate level”, and grades 5–6 designated a “high level” (Frändin and Grimby, 1994). The frequency of at least a 30-min brisk walk was estimated for the previous summer and winter seasons. Questions about smoking habits were classified as non-smoker or present/previous smoker. Twenty-four questions dealt with knowledge of osteoporosis (Waller et al., 2002). Some questions were addressed to those ≥ 65 years and dealt with safety behavior to prevent falls, and anxiety of having a fall (Grahn Kronhed et al., 2005).

A portable device the dual X-ray and laser (DXL) technology by Calscan (Demetech, Solna, Sweden), was used between January and April 2006 to measure calcaneal bone mineral density (BMD). DXL technology by Calscan performed the scan in less than one minute, with a radiation-effective dose administration of < 0.2 μSv (Martini et al., 2004, Kullenberg and Falch, 2003). The longitudinal in-vivo precision (coefficient of variation) of the Calscan device has been estimated at 1.2% (Kullenberg and Falch, 2003). The left heel was chosen as the measurement site. BMD values are presented as g/cm^2^ (Swanpalmer and Kullenberg, 2000). A Swedish reference population for DXL in women was used for the calculation of T- and *Z*-scores (BMD value adjusted for age by the reference group) (Kullenberg, 2003).

Body weight was measured on a digital balance scale (kg), and height was measured with a stadiometer (cm). Body mass index (BMI) was calculated as weight in kg/(height in metres squared). In the present study those with missing weight and height measurements had their BMI calculated from their reported values in the questionnaire. Experienced nurses at Vadstena and Kvarnholmen PHCCs carried out these measurements.

#### 2.1.3 Statistical analysis

Group characteristics were reported as means (M) and standard deviations (SD). Percentages and 95% confidence intervals (CI) were used for reported physical activity levels, falls, and safety behavior. The independent two-sample *t*-test was used for comparison between groups. Correlations between age and present height, and between calcaneal BMD and height loss, were studied by using the Pearson correlation coefficient (r). Multiple regression analysis of women and men separately was used to study independent variables with possible impact on calcaneal BMD. These variables were checked for multicollinearity. Logistic regression with odds ratios of the risk for self-reported fractures after the age of 50 were checked and adjusted for several independent variables. In the fully adjusted model, DXL T-scores dichotomised into normal/osteopenic and osteoporotic, age, BMI values, leisure time physical activity grades dichotomised into low, moderate and high levels, 30-min daily summertime brisk walks dichotomised into yes or no, and community residence dichotomised into the I- and C-groups were used for the analyses. A *p*-value < 0.05 was considered significant. Statistical computations were performed using the Statistica software (version 13). The extended Vadstena Osteoporosis Prevention Project was approved by the Regional Ethics Research Committee for Human Research, Faculty of Health Sciences, Linköping University.

A written consent was requested for inclusion in the study.

## 3 Results

A total of 417 persons (63% of those invited) consisting of 237 women and 180 men in the intervention (I) group and 120 persons (47% of those invited) consisting of 74 women and 46 men in the control (C) group underwent calcaneal measurements and completed the questionnaire (Figure 1). In the I-group a total of 337 persons (81%) were familiar with the VOPP program.

### 3.1 Characteristics of participants

The participants were born between 1910 and 1960. The mean age in the I-group was 63.5 years (range 37–94 years) and in the C-group 63.3 years (range 38–85 years). There was no difference between the I- and C-groups concerning age or BMI (Table 1). Present height was negatively correlated to age in both women (*n* = 310) (*r* = − 0.359, *p* < 0.001) and men (*n* = 226) (*r* = − 0.352, *p* < 0.001).

### 3.2 Physical activity levels

More men i.e. 52.2% (95%CI 0.443–0.601) in the I-group (157 respondents) reported a heavy work level than in the C-group 22.5% (95%CI 0.090–0.360) (40 respondents).

Most participants reported a moderate leisure-time physical activity level. There was no difference regarding physical activity levels or 30-min daily brisk walks between women or between men in the groups.

### 3.3 Bone-specific drugs

Current use of bisphosphonates due to osteoporosis was reported by only 3.8% (*n* = 9) of the women and 1.7% (*n* = 3) of the men in the I-group, and by 4.1% (*n* = 3) of the women in the C-group.

### 3.4 Questions concerning knowledge of osteoporosis

There was a total of 301 persons in the I-group, and 89 in the C-group, that answered all questions concerning knowledge about osteoporosis. A small difference was found between the groups concerning the total correctly answered questions with a mean value of 18 in the I-group, and 17 in the C-group (*p* < 0.05).

### 3.5 Fall frequency and safety behavior in persons ≥ 65 years of age

Reported falls for the previous year did not differ between the groups, with a total of 83 persons reporting a fall. Most falls had occurred outdoors, and were caused by slipping on snow or ice, while other causes were tripping on rugs or pets, stumbling on pavements or holes in the street, and bicycling accidents. Ten persons in the I-group and two persons in the C-group limited their daily activities due to a fall. Many elderly people were worried about a new fall. The reported use of shoe or cane spikes in winter was higher among elderly women in the I- than in the C-group (Table 2).

### 3.6 Calcaneal bone mineral density

There was no difference between the groups concerning calcaneal BMD values, T- and *Z*-scores for the genders (Table 1). Normal calcaneal T-scores were found in 263 persons, osteopenic values in 204 persons, and osteoporotic values in 70 persons. Calcaneal BMD was negatively correlated to height loss in both women (*n* = 169, *r* = − 0.557) and men (*n* = 132, *r* = − 0.341) (*p* < 0.001). Calcaneal BMD was negatively associated with age (*p* < 0.001), and positively with BMI (*p* < 0.001) in women (*n* = 283) and in men (*n* = 210). Calcaneal BMD was negatively associated with reported fracture in adulthood (*p* < 0.05) and positively associated with 30-min daily summertime brisk walks (*p* < 0.05) in women, and to moderate (*p* < 0.05) and high leisure time physical activity levels (*p* < 0.05) in men (Table 3). There was no problem regarding multicollinearity between the independent variables, i.e. the absolute correlation between independent variables was, in each case, below 0.7.

### 3.7 Self-reported fractures

A total of 122 participants (65 women and 22 men in the I-group and 22 women and 13 men in the C-group) reported a fracture in adulthood. There was no difference between the groups concerning fracture after the age of 50 (439 participants included, mean age 68), with 51 women and 6 men in the I-group and 16 women and one man in the C-group reporting a fracture. The fully adjusted model of logistic regression for women (*n* = 224) and men (*n* = 176), showed that participants with an osteoporotic calcaneal T-score (≤ − 2.5) had a 3.2 increased risk (95%CI 1.48–6.89) of having a history of a self-reported fracture after the age of fifty compared to those with a calcaneal T-score above − 2.5 (Table 4).

## 4 Discussion

The present evaluation of the extension of a 10-year population-based osteoporosis and fall prevention program showed only a small effect of the previous interventions. A population wide strategy was the core in the 1990s in contrast to a more high-risk strategy in the 2000s (Waller et al., 1997, Waller et al., 2002, Grahn Kronhed et al., 2006, Grahn Kronhed et al., 2005, Grahn Kronhed et al., 2004). There was somewhat greater knowledge of osteoporosis in the I-group compared to the C-group according to a questionnaire, and a difference concerning safety fall prevention behavior in elderly women in the I-group, reporting greater use of spikes in winter than the C-group. The mediaeval architecture with cobblestones squares and lanes in both city centres may have implied an increased risk of slipping and sustaining a fracture. The shoe shop in the intervention area collaborated with the program, marketing shoe spikes in winter that may have served as a good reminder (Grahn Kronhed et al., 2006, Grahn Kronhed et al., 2005). Experiences from other injury prevention projects indicate that effects of community-based interventions could be expected in the long run, preferably for a period of more than ten years (Spinks et al., 2005). The motivation and the maintenance of a changed life-style behavior to improve bone health, prevent falls and fractures in a general population is a difficult challenge (Anon, 1994).

Invited follow-up persons seemed to be more eager to participate (77.9%) than new persons in the intervention (55%) and in the control groups (47%). Mean ages in women and men in Vadstena community were 46.9 years and 43.1 years respectively, while mean ages in women and men in Kalmar community were 41.9 years and 39.4 years respectively, which are in agreement with mean ages in the total Swedish population (women M = 42.1 years and men M = 39.7 years). The proportion of community residents aged ≥ 65 years was 23% in Vadstena and 17% in Kalmar, while this proportion was 17% for all of Sweden (Anon, n.d.). A greater proportion of elderly women participated in the I-group (20% aged ≥ 80 years) than in the C-group (7% aged ≥ 80 years). A reason for this age heterogeneity was that the nurse in the intervention area brought the portable device to senior home residents to measure those who could not visit the PHCC on their own, while the nurse in the control area measured calcaneal BMD only at the PHCC.

The FRAX® tool integrates the weight of clinical risk factors for the 10-year probability of hip fracture, or a major osteoporotic fracture, in persons aged 40–90 using patient-derived clinical risk factors, with or without the inclusion of femoral neck BMD*.* Ever since the introduction of FRAX® by the Swedish National Board of Health and Welfare in 2012 clinicians are urged to use the tool to assess the need of treatment to prevent fractures in osteoporotic patients. Several health care regions have implemented FRAX to find out high-risk patients in order to give adequate medical treatment for the prevention of a secondary fracture. Some regions also use coordinated, multi-disciplinary models of care for secondary fracture prevention according to the recommendation by the International Osteoporosis Foundation (Javaid et al., 2015). However, the FRAX algorithm model does not include other risk factors such as physical inactivity and propensity to falls (Kanis and on behalf of the World Health Organization Scientific Group, 2007). The fracture probability computed by the FRAX algorithm may be underestimated with multiple fractures and with a clinical vertebral fracture, which is an especially strong risk factor and may only be detected by a radiographic observation alone. Considerable height loss may indicate a vertebral fracture according to a study by Xu et al. (Xu et al., 2011). Thirty percent of those participating in bone mineral density measurements by DXA would have been misclassified, if BMD classification had been used solely (Xu et al., 2011). The negative correlation (*r* = − 0.557) between calcaneal BMD and height loss in women in our study may provide additional information to fracture risk assessment. The calculation of odds ratios of the risk for having a fracture according to an osteoporotic calcaneal T-score probably gives valuable information to the primary health care. The present study shows a fracture-predictive ability by the osteoporotic calcaneal T-score, which is in accordance with the study by Lundin et al. In their study the DXL Calscan was as good a predictor of future fractures as DXA, including FRAX. They concluded that calcaneal DXL could be a useful tool in a clinical fracture risk assessment, if DXA equipment is not available (Lundin et al., 2015). Another Swedish study showed that low calcaneal BMD, by using DXL-technique, was related to a hip and fragility fracture risk (Albertsson et al., 2010).

The present study showed a positive association between moderate, respectively, high physical activity levels and calcaneal BMD values in men. This is partly in agreement with a previous study, which found a positive association between moderate, respectively, high physical activity levels and calcaneal stiffness (measured by quantitative ultrasound) for both genders (Grahn Kronhed et al., 2004). In the present study the importance of adding questions about daily physical activity was considered, as the used leisure time physical activity questionnaire did not emphasize daily physical activity (Frändin and Grimby, 1994). The reported daily 30-min brisk walk in summer was positively associated with calcaneal BMD in women (*p* < 0.05). The international physical activity guidelines for public health recommend at least 150 min of moderate intensity 5–7 days a week, such as brisk walks for all adults (Anon, 2009). Only 34.1% of the participants reached this level summertime and 25% wintertime. The national guidelines by FYSS 2017 emphasize the need of increased physical activity in the prevention and treatment of osteoporosis for all Swedish adults (Anon, 2016). Vadstena PHCC also implemented physical activity referrals for the last decade that recommend patients with life-style diseases to become more physically active. Muscular strength training and bone loading exercise is recommended to prevent and treat osteoporosis. There is evidence for a dose-response association, where higher physical activity level is related to lower fracture risk (Anon, 2009, Anon, 2016, Khan et al., 2001). It is a challenge to evaluate whether patients are compliant to the physical activity recommendation. There is still no ideal way of measuring targeted bone loading, though there have been attempts using physical activity questionnaires, accelerometers, or by measuring ground reaction forces (Khan et al., 2001). A therapeutic effect of increased physical activity on bone mass may be detected at a rather early stage by calcaneal bone mass measurement, as the calcaneus is mainly cancellous bone (90%), with a high metabolic turnover (Grahn Kronhed et al., 2004, Khan et al., 2001). The measurement of calcaneal BMD and the use of a diary with recorded physical activity may be one way of motivating physically inactive people to become more physically active.

### 4.1 Strengths and limitations

The long-term follow-up study presents results from an extension of a population-based osteoporosis and fall prevention program implemented in a Swedish community for a period of more than ten years. The selected areas for the study comparison were rather similar to the geography, the mediaeval architecture areas, and also the population's mean ages with many elderly in both areas.

The effect of health education on osteoporosis and fractures aimed at young people at schools and kindergartens could not be seen until later on in adult life. New participants in the intervention group might not have been influenced by the ten-year intervention, if they had recently moved to Vadstena. Community-based osteoporosis interventions might be more focused on the prevention of fractures in the elderly who are exposed to sustaining an osteoporotic fracture when ageing. Probably the community services should undertake more responsibility for public health messages and also frequently give safe fall prevention advice and offer exercise facilities for the elderly. It may be tiresome for a small project group to arouse enthusiasm to a general population for a long time period of 17 years and the project members may also be exchanged as time goes by*.* The difference in risk potential for the studied health outcome may have decreased between the groups, as national campaigns and regional osteoporosis health care programs, with targeted information on risk factors for fractures, probably influenced residents in both areas. The design of the follow-up study was cross-sectional with a new control area, as collaboration was initiated between the intervention and the former neighbouring control communities after the ten-year intervention period.

## 5 Conclusions

The long-term effects of a ten-year community-based osteoporosis intervention program on knowledge and behavior were modest seven years after its completion.

## Figures and Tables

**Table 1:** Mean (M) values and standard deviations (SD) for age, calcaneal bone mineral density (BMD), T- and *Z*-scores, body mass index (BMI), height and weight for women and men in the intervention (I) and the control (C) groups. (Vadstena; Kvarnholmen/Kalmar city centre, Sweden in 2006).

	Women			Men		
	I-group	C-group	*p*-value	I-group	C-group	*p*-value
	M (SD)	M (SD)		M (SD)	M (SD)	
Number	237	74		180	46	
Age	64.2 (14.8)	62.6 (12.0)	ns	62.5 (12.9)	64.4 (12.5)	ns
BMD (g/cm^2^)	0.394 (0.095)	0.400 (0.077)	ns	0.504 (0.084)	0.504 (0.096)	ns
T-score	− 1.5 (1.2)	− 1.4 (1.0)	ns	− 0.7 (1.1)	− 0.6 (1.2)	ns
Z-score	− 0.3 (0.9)	− 0.3 (0.7)	ns	0.3 (1.0)	0.3 (1.2)	ns
Number	236	74		180	46	
BMI kg/(m)^2^	26.3 (4.7)	25.7 (3.9)	ns	27.3 (4.3)	26.7 (3.6)	ns
Number	233	74		178	46	
Height (cm)	163.4 (6.4)	163.8 (6.8)	ns	176.3 (6.6)	178.5 (8.2)	ns
Weight (kg)	70.1 (12.0)	69.2 (11.8)	ns	85.1 (15.0)	85.2 (13.8)	ns

**Table 2:** Reported falls, limitations due to a fall, fear of falling, and the use of spikes in percent and confidence intervals (CI) with 95% confidence level for each question in women and men aged ≥ 65 years in the intervention (I) and the control (C) groups. (Vadstena; Kvarnholmen/Kalmar city centre, Sweden in 2006).

	WOMEN		MEN	
	I-group	C-group	I-group	C-group
I had a fall last year				
Total respondents (n)	105	36	72	21
Percent and CI	39% (0.296–0.485)	27.8% (0.124–0.431)	30.6% (0.197–0.415)	33.3% (0.113–0.553)

I have limited my daily activities due to a fall				
Total respondents (n)	46	11	24	8
Percent and CI	13% (0.033–0.228)	9.1% (− 0.079–0.261)	16.7% (0.018–0.316)	12.5% (− 0.104–0.354)

I am worried about falling				
Total respondents (n)	47	13	26	8
Percent and CI	66% (0.524–0.795)	46.2% (0.191–0.733)	34.6% (0.163–0.529)	37.5% (0.040–0.710)

I use spikes in winter when it is slippery				
Total respondents (n)	106	37	71	21
Percent and CI	67% **(0.580–0.759)**	40.5% (0.247–0.564)	29.6% (0.190–0.402)	23.8% (0.056–0.420)

The difference between the groups is significant, if the 95% confidence intervals for women and men respectively do not overlap.

**Table 3:** Regression coefficients for selected independent variables (age, BMI, leisure time physical activity levels, 30-min daily brisk walks in summer, sustained fractures in adulthood and community) and their associations with calcaneal bone mineral density (BMD) in women (*n* = 283) and men (*n* = 210) (Vadstena; Kvarnholmen/Kalmar city centre, Sweden in 2006).

	BMD women		BMD men	
	β-coefficients	*P*-value	β-coefficients	*P*-value
Intercept	0.551	**0.000**	0.446	**0.000**
Age (years)	− 0.004	**0.000**	− 0.002	**< 0.001**
BMI (kg/m^2^)	0.004	**< 0.001**	0.006	**< 0.001**
Moderate leisure time activity	0.013	0.234	0.032	**0.032**
High leisure time activity	0.017	0.385	0.059	**0.013**
30-min daily brisk walks in the summer	0.018	**0.020**	0.016	0.215
Fractures in adulthood	− 0.023	**0.012**	− 0.030	0.061
Community	− 0.002	0.797	− 0.009	0.537

**Table 4:** Odds ratios (OR) and 95% confidence intervals of the risk for self-reported fracture after the age of 50 in women (*n* = 224) and men (*n* = 176). Two models were used and adjusted for several independent variables. Patients with missing data on BMI, leisure time physical activity level, 30-min daily brisk walks in summer were excluded from the analyses. (Vadstena; Kvarnholmen/Kalmar city centre, Sweden in 2006).

Variable	Crude OR	Model 1: Age- and sex adjusted	Model 2: Fully adjusted
DXL T-score			
Normal/Osteopenia	1.0 (Reference)	1.0 (Reference)	1.0 (Reference)
Osteoporosis	**8.14** (4.45–14.88)	**2.88** (1.38–5.98)	**3.20** (1.48–6.89)
*P*-value	*p* = 0.000	*p* = 0.005	*p* = 0.003
Age		**1.06** (1.03–1.10)	**1.06** (1.03–1.10)
*P*-value		*p* < 0.001	*p* < 0.001
Sex			
Men		1.0 (Reference)	1.0 (Reference)
Women		**7.48** (3.22–17.40)	**7.42** (3.19–17.30)
*P*-value		*p* < 0.001	*p* < 0.001
BMI (kg/m^2^)			1.03 (0.97–1.11)
*P*-value			*p* = 0.337
Leisure time physical activity			
High activity			1.0 (Reference)
Low activity; *P*-value			1.26 (0.13–11.89); *p* = 0.915
Moderate activity; *P*-value			1.38 (0.17–11.50); *p* = 0.725
30-min daily brisk walks in summer			
Yes			1.0 (Reference)
No			1.02 (0.53–1.95)
*P*-value			*p* = 0.951
Community			
Intervention group			1.0 (Reference)
Control group			1.20 (0.60–2.40)
*P*-value			*p* = 0.612
